# A case for combined environmental stressor studies

**DOI:** 10.1186/2046-7648-1-7

**Published:** 2012-10-09

**Authors:** Michael Tipton

**Affiliations:** 1 Extreme Environments Laboratory, Department of Sport and Exercise Science, University of Portsmouth, Spinnaker Building, Cambridge Road, Portsmouth PO1 2ER, UK

**Keywords:** Environmental stress, Cold, Hypoxia, Heat, Microgravity, Integrative physiology

## Abstract

This commentary considers the reasons for the relative dearth of studies examining combinations of environmental stressors and makes a case for more of these studies.

## Background

It is a reason for celebration that in *Extreme Physiology and Medicine*, we have, within the pages of one journal, high quality papers focused on the full range of stressors that constitute the environment: from hot to cold, from microgravity to hyperbaric. However, it still remains something of a mystery why so little effort or funding appears to have been given to studying these stressors in combination.

## Main text

It seems ironic that those of us who eulogise about the importance and benefits of studying integrative whole body physiology refuse to practise what we preach when it comes to environmental stressors. So it is that people rightly emphasise the connectivity and co-dependence of the major systems of the body and then say, “I work in hypoxia”. In part, this is driven by funding, by interest, by facility availability and by an education system that teaches us more and more about less and less, and pigeon holes our “expertise”, thereby reinforcing the above and limiting the opportunity to diversify.

## Discussion

The problem is that in the “real world”, things are not like that. Cold and hypoxia usually co-exist, as do cold and hyperbaria. In deserts, heat and cold alternate. Our athletes, our military, some occupational groups and many outdoor pursuit enthusiasts seldom encounter environmental stressors in isolation. Table 1 shows the studies undertaken between 1948 and 2012 that can be described as examinations of combinations of environmental stressors (articles written in English and listed in PubMed and Google scholar). The limited extent of this table highlights the paucity of work in this area over the last 65 years.

**Table 1:** Combined environmental stressor research papers 1948 to 2012 (heat and humidity not considered as a “combination”)

**Environmental stressors**	**Animal/human**	**Number of articles**
Hypoxia and cold	Human	13
Hypoxia and cold	Animal	49
Hypoxia and heat	Human	1
Hypoxia and heat	Animals	3
Bed rest and hypoxia	Human	3
Bed rest and heat	Human	3
Bed rest and cold	Human	1
Cross adaptation heat and hypoxia	Human	1
Cross-adaptation heat and hypoxia/ischaemia	Animals	7
Cross-adaptation cold and hypoxia	Human	1
Cross-adaptation cold and hypoxia	Animals	4

It can be concluded that few laboratory studies have examined, for example, the combined effects of altitude and cold or heat, e.g*.*[1], or microgravity and hypoxia and hypercapnia, e.g. [2], using either sequential or simultaneous exposures. The exception to this is heat and humidity which, because of their combined impact on heat loss, have always been considered in combination.

Some may claim that investigating how factors like exercise, high environmental temperature and clothing affect the physiology of the body and, as a consequence, performance qualifies as the study of combined stressors; certainly, this kind of research is prevalent. However, of course, only one of these is an environmental stressor. Others may argue that they do not study combined stressors because they want to understand the “mechanism” of a particular response and are concerned that combining stressors will confuse and prevent understanding. However, this concern could be addressed by suitable experimental design; indeed, such a design may reveal that some responses only occur, or are more likely, when combined stressors are present, e.g. [3].

## Conclusions

Hence, for the sake of those who go and work and play around the globe, and have to perform when confronted with combined environmental conditions, shouldn't we give a little more attention to understanding how co-existent environmental stressors affect the physiological and pathophysiological responses of the body?

## Competing interests

The author declares that there are no competing interests.
